# Development and Broad Application of a Double Drop-Off ddPCR Assay for Simultaneous Detection of Four *FGFR3* Mutations in Tissue and Liquid Biopsy Samples

**DOI:** 10.3390/cancers18162634

**Published:** 2026-08-14

**Authors:** Eleni Thanou, Nikos Gavalas, Eleni Kabrani, Foteini Grigoriou, Anna Konstantinou, Vasiliki Malamatini, Christina Chourdaki Peristeri, Evi Lianidou, Aristotelis Bamias, Athina Markou

**Affiliations:** 1 Analysis of Circulating Tumor Cells, Laboratory of Analytical Chemistry, Department of Chemistry, University of Athens, 15771 Athens, Greece; elenathanou@chem.uoa.gr (E.T.); lianidou@chem.uoa.gr (E.L.); 2 2nd Propaedeutic Department of Internal Medicine, Attikon University Hospital, National & Kapodistrian University of Athens, Chaidari, 12462 Athens, Greece; ngavalas@med.uoa.gr (N.G.); grigorioufoteini4@gmail.com (F.G.); annakonstantinoumed@gmail.com (A.K.); malamatinivas@gmail.com (V.M.); pechouchristi@gmail.com (C.C.P.); abamias@med.uoa.gr (A.B.); 3 Laboratory of Molecular Pathology, Department of General and Anatomic Pathology, Aristotle University School of Medicine, 54124 Thessaloniki, Greece; kabrani.eleni@gmail.com

**Keywords:** FGFR3 mutations, drop-off ddPCR, bladder cancer, liquid biopsy, erdafitinib

## Abstract

Background: Fibroblast Growth Factor Receptor 3 (*FGFR3*) mutations are common and clinically relevant alterations in bladder cancer, with implications for diagnosis, monitoring, and patient selection for targeted therapies. Tissue-based testing is often limited by sample availability, invasiveness, and the need for repeated sampling. This study aimed to develop and analytically validate a sensitive double drop-off droplet digital polymerase chain reaction (ddPCR) assay for simultaneous detection of four FGFR3 hotspot mutations in tissue and liquid biopsy samples. Methods: The assay targeted four *FGFR3* mutations (S249C, R248C, Y373C, G370C) using reference probes that generate a constant fluorescence signal and wild-type-specific drop-off probes that lose binding when a mutation is present, thereby distinguishing wild-type double-positive droplets from mutant droplets with reduced drop-off fluorescence. Analytical validation was performed using synthetic mutant oligonucleotides, wild-type genomic DNA, and cell-free DNA (cfDNA) from healthy donors (HDs). Specificity, limit of blank (LOB), limit of detection (LOD), and assay precision were evaluated. Performance was compared with next-generation sequencing (NGS) in formalin-fixed paraffin-embedded (FFPE) tissue DNA. *FGFR3* mutations were also assessed in matched plasma and urinary cfDNA from bladder cancer patients. Results: The assay demonstrated clear cluster separation, no cross-reactivity, and reliable detection of all mutations down to 0.2% mutant allele frequency (MAF). Strong agreement was observed with a mutation-specific singleplex ddPCR assay for S249C. Concordance with NGS in tissue DNA was 74.2%, with ddPCR identifying additional low-abundance mutations not reported by NGS. *FGFR3* mutations were detected in plasma and urinary cfDNA, with complete concordance between matched plasma and urine samples. Conclusions: This ddPCR assay provides a rapid, sensitive, and cost-effective method for detecting clinically relevant *FGFR3* mutations and may complement sequencing-based approaches for molecular monitoring using tissue, plasma, and urine specimens.

## Simple Summary

Bladder cancer is a common disease worldwide, and alterations in the *FGFR3* gene are important because they can indicate patients who may benefit from targeted treatments. However, current testing often depends on tumor tissue availability, which may be difficult to obtain, especially when repeated monitoring is needed. In this study, we developed a sensitive laboratory test that can detect four common *FGFR3* mutations at the same time using very small amounts of DNA. The test was evaluated in tumor tissue, blood, and urine samples from patients with bladder cancer. Our findings show that this method can detect low levels of *FGFR3* mutations, including some that may be missed by broader sequencing methods. This approach may provide researchers and clinicians with a rapid, cost-effective, and less invasive tool for studying *FGFR3* alterations and improving molecular monitoring in bladder cancer.

## 1. Introduction

Bladder cancer (BC) ranks among the ten most common malignancies worldwide [1] and represents the most common urothelial cancer (UC), which can affect any part of the urinary tract. Although most patients present with non-muscle-invasive bladder cancer (NMIBC), 25% present with muscle-invasive bladder cancer (MIBC) and 5% with upfront metastatic disease (mBC). In addition, approximately 30% of NMIBCs progress to muscle-invasive disease. Treatment of bladder cancer depends on disease stage. Non-muscle-invasive disease is generally managed with transurethral resection followed, when appropriate, by intravesical therapy, whereas muscle-invasive disease is treated with radical cystectomy and perioperative systemic therapy. Advanced or metastatic disease may require chemotherapy, immunotherapy, antibody–drug conjugates, or targeted therapy such as erdafitinib for tumors harboring susceptible *FGFR3* alterations [2]. MIBC is associated with significant mortality from bladder cancer and a greater mutational burden, involving *TERT*, *TP53*, *PIK3CA*, *STAG2*, chromatin-modifying genes, and particularly *FGFR3* [3,4,5,6,7,8]. Activating *FGFR3* mutations occur in ~50% of bladder cancers, compared with ~1–2% of most other solid tumors, and are especially frequent in low-grade tumors, reaching 60–70% [9,10,11].

Nine recurrent *FGFR3* mutations have been identified across exons 7, 10, and 15, with S249C located in exon 7 being the most prevalent one, accounting for ~62% of cases. *FGFR3* mutations are somatic activating alterations acquired during urothelial tumorigenesis. Although their exact etiology remains unclear, their development may be associated with accumulated DNA damage from carcinogenic exposures, such as tobacco smoke and occupational chemicals, as well as endogenous mutational processes [12]. *FGFR3* alterations are routinely studied as a predictive biomarker for metastatic mUC to guide treatment with erdafitinib, an FDA-approved pan-FGFR kinase inhibitor [13,14]. Its FDA-approved companion diagnostic, the Therascreen FGFR RGQ RT-PCR assay, detects selected *FGFR3* mutations and fusions in formalin-fixed, paraffin-embedded (FFPE) urothelial tumor tissue. Although tissue-based RT-PCR and NGS assays have improved molecular stratification, they require invasive sampling, which can be challenging in patients with limited tissue, advanced disease, or repeated transurethral resection of bladder tumors (TURB-T).

Liquid biopsy (LB) has emerged as an attractive alternative approach for non-invasive disease assessment. LB enables longitudinal monitoring of cancer progression through the analysis of tumor-derived biomarkers in body fluids, including blood, urine, and saliva [15,16,17]. In bladder cancer, urine represents a particularly relevant liquid biopsy source because of its direct anatomical contact with the tumor, while plasma-based approaches may provide complementary information, especially in advanced or metastatic disease. Furthermore, urine is easier to obtain than plasma, especially for monitoring therapeutic results, requiring repeated samples.

Several clinically integrated molecular platforms have been developed for BC detection and monitoring [18]. Urine-based assays such as UroSEEK detect alterations across 11 key genes, capturing common genetic drivers of the disease [19,20], while OncoUrine combines multiplex PCR-based NGS for *FGFR3* variants with a 17-gene panel and ONECUT2 methylation analysis in preoperative urine samples [21]. Although these approaches provide broad molecular information, they often require NGS workflows and specialized bioinformatics, limiting their suitability for rapid, cost-effective monitoring of selected recurrent mutations. PCR-based assays, including Uromonitor, a qPCR test targeting *TERT*, *FGFR3*, and *KRAS* mutations, have shown promising analytical performance [22], and recent ddPCR-based evaluation of the same targets in urine demonstrated higher sensitivity than qPCR and cytology [23]. However, current PCR- and ddPCR-based *FGFR3* assays generally interrogate only a restricted number of predefined hotspot mutations, often a single one or a narrow mutation set per assay. This limits clinical implementation, as simultaneous detection of multiple clinically relevant *FGFR3* hotspots could improve assay efficiency, sample conservation, and applicability in low-input liquid biopsy specimens.

Our group has previously developed drop-off ddPCR assays for the simultaneous detection of ten *PIK3CA* mutations in plasma samples [24] and five of the most common CXCR4 mutations in the bone marrow [25]. The drop-off ddPCR is an efficient tool to screen for clustered mutations that can be covered by a single TaqMan probe as it is based on the design of a specific reference probe, which provides a consistent signal, along with a wild-type (WT) “drop-off” probe designed not to bind when a mismatch is present in the gene sequence [26,27]. Consequently, WT samples generate a double-positive fluorescence signal, whereas the presence of a mutation is confirmed by the loss of the drop-off signal, resulting in a single-positive signal [24,25,26,27].

In this context, the present study aimed to develop and analytically validate a highly sensitive and specific multiplex drop-off ddPCR assay for the simultaneous detection of the four most frequent *FGFR3* mutations associated with bladder cancer. By combining multiplex detection with the high analytical sensitivity of ddPCR, this approach overcomes the limitations of highly targeted single-mutation PCR assays and provides a robust, rapid, and cost-effective tool for *FGFR3* mutation screening. Importantly, the assay was developed with compatibility for both tissue-derived DNA and liquid biopsy components, supporting its potential implementation in non-invasive molecular profiling and longitudinal disease monitoring. Finally, we applied the assay to paired urine and plasma samples and included a subset of matched tissue samples previously analyzed through independent molecular testing, enabling the evaluation of concordance across different biological sample types and further supporting the clinical applicability of this approach.

## 2. Materials and Methods

### 2.1. Patients

Twenty-five patients with histologically confirmed BC of any stage who had not undergone cystectomy were included in the study. The protocol of the study was approved by the Institutional Review Board (IRB) of ATTIKON Hospital and all patients provided their informed consent for sample collection and analysis of clinical data. Demographic and clinical characteristics of included patients are shown in Supplementary Table S1. As this was an analytical assay development study, available clinical samples meeting the eligibility criteria were included to evaluate the applicability of the method in tissue and liquid biopsy specimens.

### 2.2. Sample Collection

Peripheral blood samples were collected in ethylenediaminetetraacetic acid (EDTA) tubes (n = 25, 2 mL), along with corresponding urine samples (n = 24, 40 mL). Samples from 24 healthy donors were also included, of which 18 were used for urine cfDNA isolation and 6 for plasma cfDNA isolation. Peripheral blood was processed within 2–4 h of collection by centrifugation at 530× *g* for 10 min at room temperature (RT), followed by a second centrifugation at 2000× *g* for 10 min. Plasma was then transferred into 2 mL tubes and stored at −70 °C until further use. Urine samples were processed within 2–4 h of collection by centrifugation at 4000× *g* for 10 min at RT, and then urine conditioning buffer (Zymo Research, California, USA) was added. Urine was then stored at RT until further use.

Thirty-one FFPE carcinoma samples were also collected for comparative analysis. Among these, corresponding plasma and urine samples were available for four patients. All patients provided written informed consent for the collection and analysis of their biological samples, in accordance with the Declaration of Helsinki.

### 2.3. Extraction of cfDNA

cfDNA was isolated from both plasma and urine samples using specific extraction protocols. Plasma cfDNA was recovered from 2.0 mL of plasma using the QIAamp Circulating Nucleic Acid Kit (Qiagen, Germany), while urinary cfDNA (ucfDNA) was extracted from 40 mL of urine using the Quick-DNA Urine Kit (Zymo, USA). Both procedures were performed according to the manufacturers’ instructions, with the final DNA eluted in a volume of 40 μL using the respective elution buffers provided in each kit.

### 2.4. Targeted NGS of FFPE Tumor Tissue

Molecular profiling of FFPE tissue samples was carried out using a targeted NGS panel (Oncomine Precision Assay, ThermoFisher Cat. No. A46291) and the automated platform of the Genexus^TM^ Dx Integrated Sequencer (IVDR) (ThermoFisher, Cat. No A53579). Hematoxylin and Eosin (H&E) sections from all tissue blocks were reviewed by a histopathologist for tumor tissue adequacy. The tumor area was assessed for tumor cellularity and necrosis and 8 µm thick sections were deparaffinized in xylene and rehydrated through ethanol for downstream nucleic acid extraction. In order to enrich the molecular template with tumor genetic material, deparaffinized sections were manually macrodissected, whenever required, according to the pathologist’s overview. Tumor cellularity ranged from 10% to 80%. Sections were digested overnight with Proteinase K at 55 °C, and on the following day nucleic acid extraction was performed using the MagMAX™ FFPE DNA/RNA Ultra Kit (Applied Biosystems, Cat. No. A31881) according to the manufacturer’s instructions. Quantification of DNA and RNA was performed using the Qubit™ 4 Fluorometer (ThermoFisher Cat. No. Q33238). As input for library preparation, 10 ng of DNA was used for all samples, and sequencing was performed using the GX5™ Chip and coupler, which generates approximately 12–15 million reads per lane. Eight different libraries are multiplexed per chip lane; variant metrics are generated through the Ion Reporter™ Software Analysis Pipeline Version 5.16 (ThermoFisher, MAN0019148). An “absence” call by the NGS analysis pipeline indicates either a variant allele frequency (VAF) of 0%, a VAF below the assay’s validated limit of detection (LOD; estimated at 1% VAF for variants supported by a sequencing depth of 4000 reads), or insufficient evidence to support confident variant calling based on predefined quality metrics, including low Phred quality score and/or *p*-value. For the discordant samples, total read coverage across the amplicon containing the corresponding SNV ranged from 1031× to 9725×.

### 2.5. Positive Controls for Drop-Off ddPCR

Synthetic oligonucleotides were used as positive controls for each mutation [gBlocks by Integrated DNA Technologies (Coralville, IA, USA)], and there was also the corresponding WT synthetic oligonucleotide (Supplementary Tables S2 and S3). Human gDNA from male (G1471; Promega, Madison, WI, USA) was used for the evaluation of LOB.

### 2.6. Double Drop-Off ddPCR Design and Data Analysis

The double drop-off ddPCR assay was developed for the simultaneous detection and quantification of four *FGFR3* hotspot mutations across two exons. Using Primer Premier 5.0 software, specific primer pairs were designed to amplify a 159 bp region in exon 7 (covering S249C and R248C mutations) and a 127 bp region in exon 10 (covering G370C and Y373C mutations). Reference and drop-off probes were also designed for each region (Supplementary Table S2).

The ddPCR analysis was conducted using the QX600 AutoDG Droplet Digital PCR System (Bio-Rad, USA). All preparation steps were performed in a dedicated pre-PCR environment using a specialized PCR hood. Each 20 μL reaction contained 10 μL of 2× ddPCR Supermix for probes (no dUTP), 750 nmol/L of each primer, and 250 nmol/L of each TaqMan probe, with a total input of up to 10 ng of cfDNA. Thermal cycling was performed on a C1000 Touch Thermal Cycler (Bio-Rad, USA) under the following parameters: 95 °C for 10 min, followed by 40 cycles of 94 °C for 30 s and 66 °C for 45 s, with a final deactivation step at 98 °C for 10 min. A ramp rate of 1 °C/s was applied throughout. Every run included no-template controls (NTC), WT controls, and positive controls. Following amplification, droplets were processed and analyzed using the QX600 Droplet Reader.

The quantification of mutant DNA copies was estimated using QuantaSoft software (Bio-Rad, USA), based on Poisson distribution statistics. Droplet populations were defined manually according to their fluorescence amplitudes in a two-dimensional (2D) plot. WT sequences were identified by FAM+/HEX+ double-positive droplets, while mutant sequences were characterized by a distinct secondary cluster with lower fluorescence amplitude in the respective region. To determine the status of each sample, a percentage score was calculated using the following formula: [HEX-FAM droplets/(FAM + HEX droplets)] × 100 [24].

## 3. Results

### 3.1. Development and Analytical Validation of the Double Drop-Off ddPCR Assay for FGFR3 Mutations

**Figure 1:**
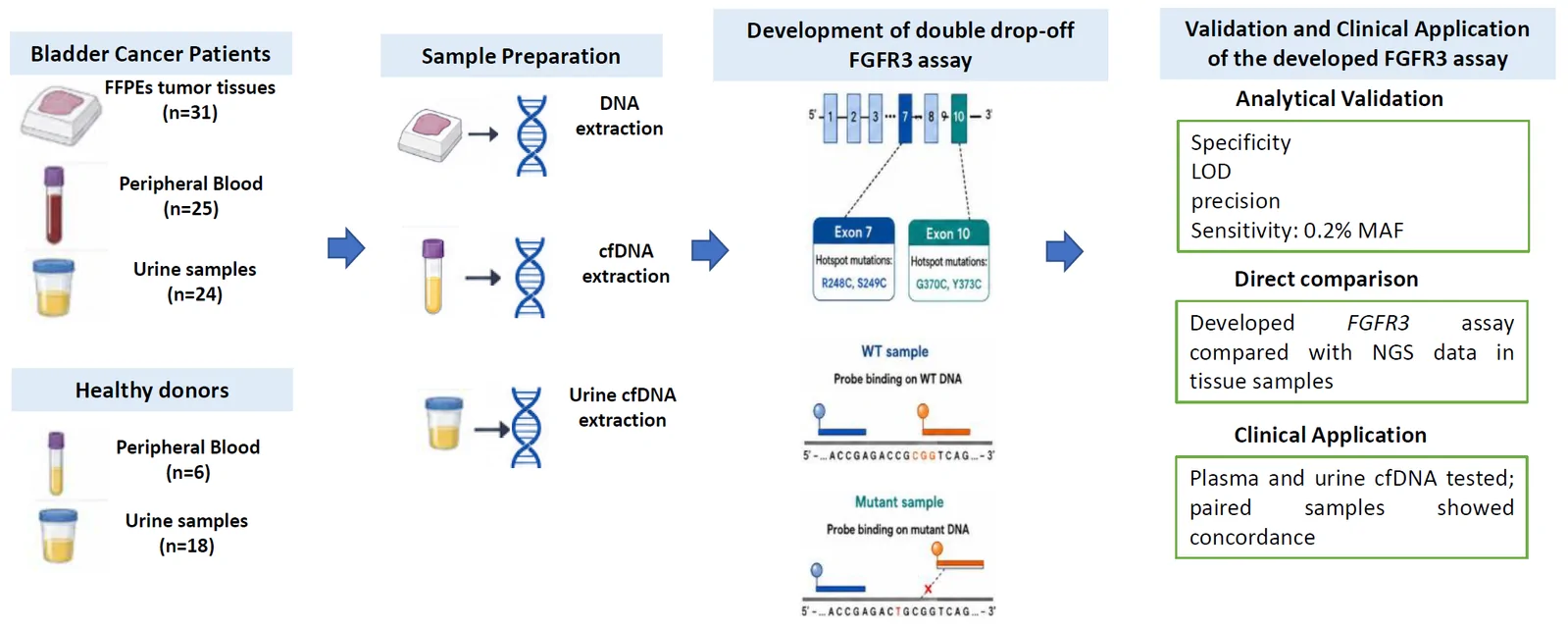
The experimental flowchart of the study.

The new drop-off ddPCR mutation assay was optimized for four mutations of *FGFR3* located in exon 7 and exon 10. The experimental flowchart of this study is presented in Figure 1.

#### 3.1.1. Optimization of Double Drop-Off ddPCR

Assay conditions were systematically optimized to maximize specificity, improve cluster separation, and reduce droplet “rain.” First, annealing temperature was evaluated across 60–67 °C, with 66 °C providing the clearest separation between positive and negative droplet clusters and minimal rain. Annealing time was then assessed, and 45 s was selected over longer incubation times of 50 s and 1 min. Further refinement of the thermal protocol included reducing the ramp rate to 1 °C/s, which additionally improved cluster resolution.

Next, primer and probe concentrations were optimized. Primer concentrations of 500, 750, and 900 nmol/L were tested, with 750 nmol/L selected as optimal. Probe concentrations were subsequently evaluated using FAM- and HEX-labeled probes at 200, 250, and 300 nmol/L, and 250 nmol/L was chosen as the final concentration. Based on these results, all subsequent ddPCR experiments were performed using an annealing temperature of 66 °C for 45 s, a ramp rate of 1 °C/s, 750 nmol/L primers, and 250 nmol/L probes.

#### 3.1.2. Analytical Validation

The optimized assay was further analytically validated using: (a) synthetic DNA oligonucleotides, (b) human male gDNA (Promega Corporation, USA), and (c) WT cfDNA extracted from the urine and plasma of healthy donors. Analytical specificity, analytical sensitivity in terms of the LOD and LOB, and intra- and inter-assay repeatability were evaluated.

### 3.2. Analytical Specificity

**Figure 2:**
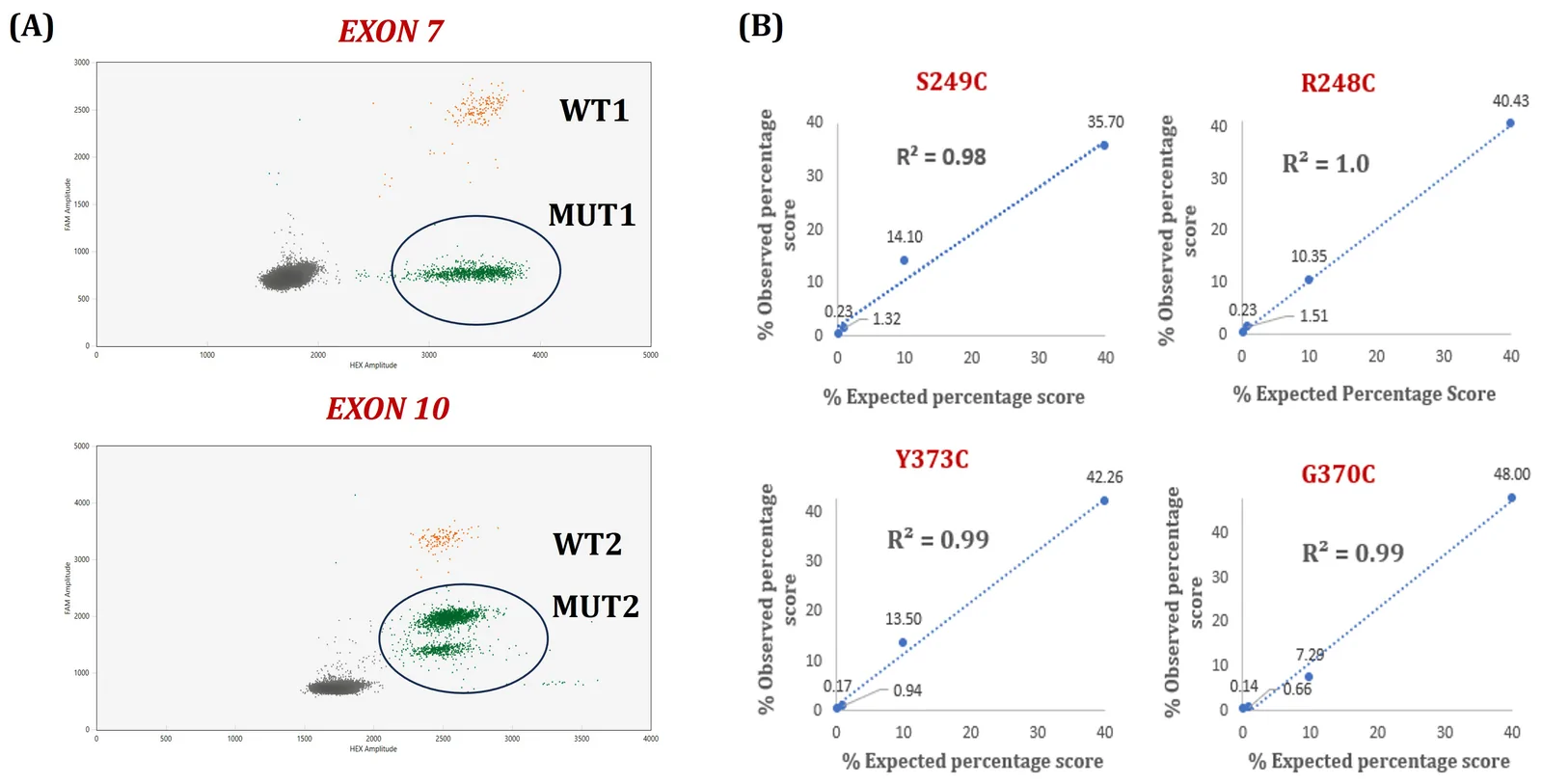
Analytical validation of the double drop-off ddPCR assay. (**A**) Representative fluorescence plots showing detection of wild-type and mutant FGFR3 alleles. (**B**) Analytical sensitivity for exon 7 and exon 10 mutations.

To evaluate analytical specificity, synthetic oligonucleotides corresponding to the S249C, R248C, Y373C, and G370C mutations were diluted in WT DNA. For each mutation tested, the assay generated a distinct secondary droplet cluster at the expected lower fluorescence amplitude, confirming the accurate detection of each specific mutation. Furthermore, no cross-reactivity or nonspecific binding among the oligonucleotides was observed (Figure 2A). In the two-dimensional plots of the four individual ddPCR reactions, the mutant droplet cluster was clearly separated from the WT cluster detected by the reference probe.

### 3.3. Analytical Sensitivity

To determine the LOB (%), cfDNA samples obtained from the urine of eighteen HDs and plasma cfDNA from six HDs were analyzed. The LOB (%) was calculated as the mean percentage of background noise plus two standard deviations (mean + 2SD), rather than based on absolute copy number, providing the expected 95%representing the expected upper limit of background signal. For exon 7, background percentage scores ranged from 0% to 0.35%, with a mean percentage score of 0.07%, resulting in an LOB of 0.33%. For exon 10, scores ranged from 0% to 0.18%, with a mean percentage score of 0.02%, establishing an LOB of 0.13%. Additionally, the LOB was calculated for Human Genomic DNA: Male DNA (Promega, USA), where no mutant droplets were detected. Any ddPCR quantification below these specific thresholds was categorized as “non-detected.”

Analytical sensitivity was then assessed using serial dilutions of synthetic mutant oligonucleotides in a WT DNA background at ratios of 40%, 10%, 1%, 0.2%, and 0%, maintaining a constant total DNA input of 10 ng per reaction. Under these conditions, the assay demonstrated high sensitivity, with a reliable detection limit of 0.2% for all targeted *FGFR3* mutations (Figure 2B). At a detection threshold of 0.1%, true positive droplets cannot be reliably discriminated from background noise, making the results unreliable.

The empirically estimated background thresholds were 0.33% for exon 7 and 0.13% for exon 10. Mutant signals were observed at the lowest tested mutant allele fraction of 0.2% for all four variants. However, because the exon 7 background threshold exceeded 0.2%, this concentration cannot be considered a formally validated LOD for exon 7. Accordingly, 0.2% is reported as the lowest tested concentration rather than the definitive assay LOD.

### 3.4. Intra- and Inter-Assay Precision

The repeatability (intra-assay precision) of the double drop-off ddPCR assay was evaluated by analyzing mutant positive controls for exon 7 and exon 10 within the same run in quadruplicate (n = 4) and triplicate (n = 3), respectively. For the exon 7 mutant positive control, the coefficient of variation (CV) was 1.87%, while for the exon 10 mutant positive control, the CV was 5.63% (Supplementary Figure S1A).

To evaluate the reproducibility (inter-assay precision), the mutant positive controls were analyzed across independent runs. The exon 7 control was tested across four separate runs (n = 4), yielding a CV of 13.6%. The exon 10 control was evaluated across three separate runs (n = 3), resulting in a CV of 9.14% (Supplementary Figure S1B).

### 3.5. Comparison of Double Drop-Off ddPCR Assay and Singleplex ddPCR Performance for S249C Detection

To investigate whether the multiplex format affected assay sensitivity and quantitative performance, S249C detection was compared between the developed double Drop-Off ddPCR Assay and a mutation-specific singleplex ddPCR assay. S249C was selected for this comparison because it represents the most commonly reported *FGFR3* hotspot mutation. This evaluation was performed using synthetic mutant oligonucleotides diluted in WT DNA at four different mutant allele frequencies in triplicate. The two assays showed a strong positive correlation across the tested dilution range, as demonstrated by Pearson correlation analysis (r = 0.991, *p* = 0.009) and a coefficient of determination of R^2^ = 0.98. Bland–Altman analysis demonstrated a mean bias (double drop-off minus singleplex) of 17.08, with 95% limits of agreement ranging from −46.72 to 80.88 (Supplementary Figure S2).

### 3.6. Direct Comparison Study Between the Double Drop-Off ddPCR Assay and NGS Profiling in FFPE Tissue DNA

**Figure 3:**
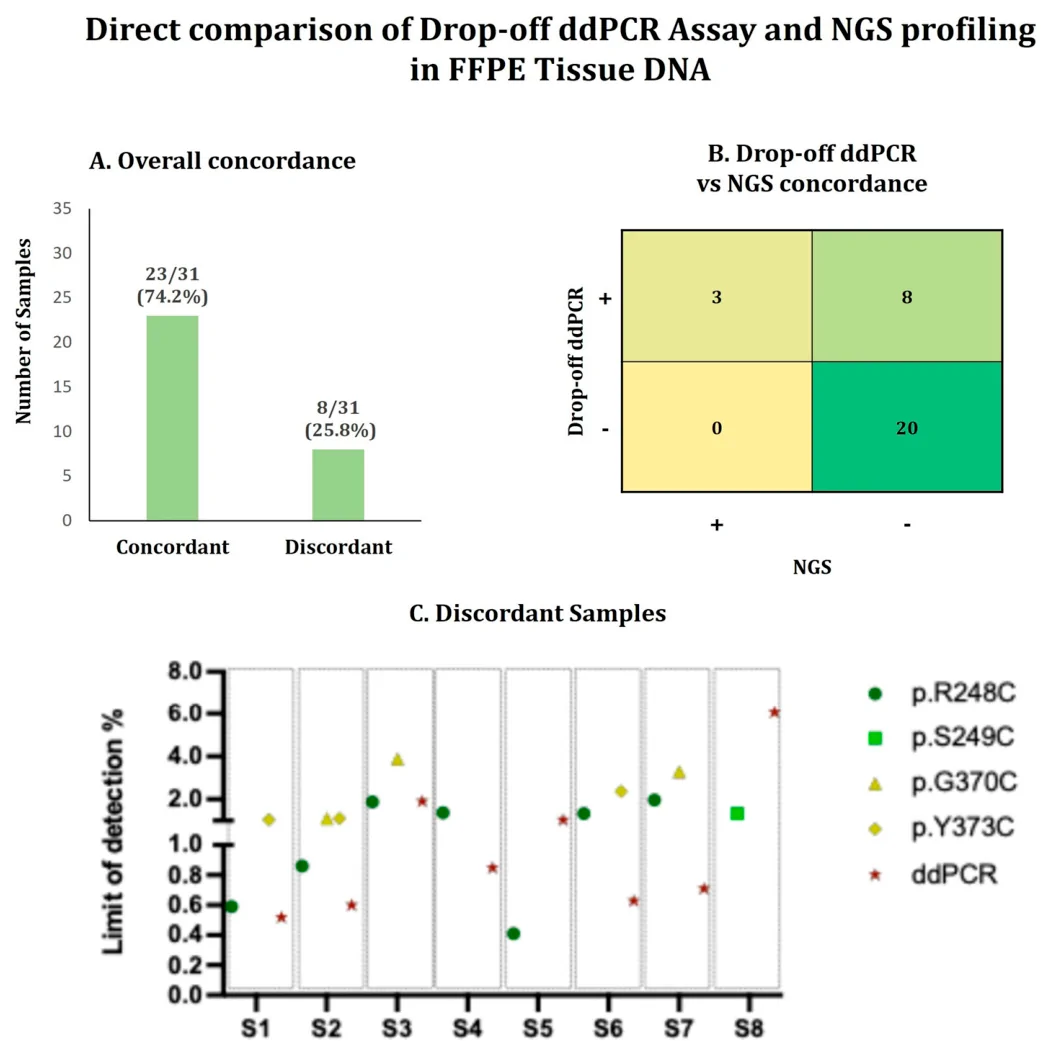
Comparison of double drop-off ddPCR and NGS in FFPE tissue samples. (**A**) Overall concordance. (**B**) Classification of concordant and discordant cases. (**C**) Mutant allele frequencies detected by ddPCR and NGS. Limit of detection as calculated by ddPCR is plotted in red.

To evaluate the performance of the developed assay in clinical tissue samples, 31 tissue DNA samples from patients previously profiled for FGFR3 mutations by NGS were analyzed. An overall observed concordance of 74.2% was observed between the two methodologies, with concordant results in 23 of 31 samples and 25.8% (8/31) showing discordant results between the two methodologies (Figure 3A). Specifically, 20 samples were classified as negative and 3 samples as positive by both assays. Using the double drop-off ddPCR assay, FGFR3 mutations were detected in 11/31 (35.5%) samples, whereas NGS detected FGFR3 mutations in 3/31 (9.7%) samples (Figure 3B). Cohen’s kappa analysis showed fair agreement between the two methods (κ = 0.326). McNemar’s exact test demonstrated a statistically significant asymmetry in discordant classifications (*p* = 0.008), indicating that the double drop-off ddPCR assay identified significantly more mutation-positive samples than NGS.

The remaining eight cases were discordant; all were identified as mutation-positive by the double drop-off ddPCR assay but classified as negative by NGS. As shown in the graph, ddPCR demonstrates a lower cumulative LOD than NGS in most cases, making it possible to detect variants present at very low allele frequencies. The NGS reporting threshold was estimated to be 1% at a sequencing coverage of 4000 reads (Figure 3C).

### 3.7. Detection of FGFR3 Mutations in Matched Tissue and Liquid Biopsy Samples from Bladder Cancer Patients

**Figure 4:**
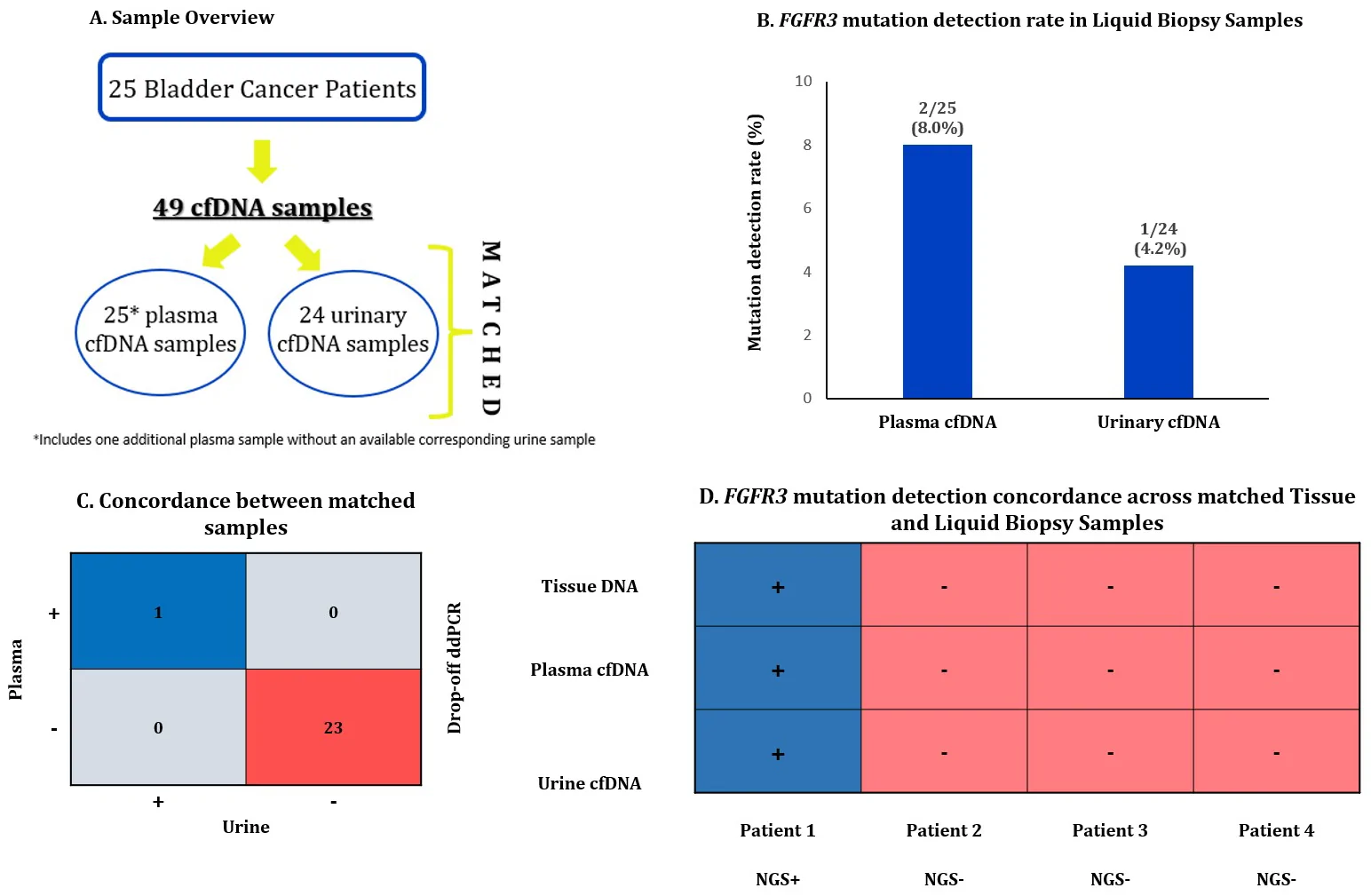
Detection of FGFR3 mutations in tissue and liquid biopsy samples. (**A**) Sample distribution and mutation detection rates. (**B**) Concordance between matched plasma and urine samples. (**C**,**D**) Results in matched tissue, plasma, and urine samples.

To evaluate the clinical applicability of the optimized double drop-off ddPCR assay in liquid biopsy samples, we analyzed 49 cfDNA samples from a bladder cancer cohort, comprising 24 matched plasma cfDNA and urinary cfDNA samples, as well as one additional plasma cfDNA sample for which no corresponding urine sample was available. *FGFR3* exon 7 mutations were detected in 2/25 (8.0%) plasma cfDNA samples and in 1/24b (4.2%) urine samples (Figure 4A). The positive urine sample corresponded to one of the two plasma-positive cases, whereas the matched urine sample for the second plasma-positive case was not available for analysis. *FGFR3* exon 10 mutations were detected in only 1/25 (4.0%) plasma cfDNA samples, for which no matched ucfDNA sample was available. Notably, one plasma cfDNA sample harbored mutations in both exons 7 and 10. Among the 24 matched plasma–urine pairs, complete concordance was observed between the two specimen types, with one patient testing positive and 23 patients testing negative in both plasma and urine (Figure 4B). Cohen’s kappa analysis demonstrated perfect agreement between plasma and urine (κ = 1.000). Consistently, McNemar’s exact test showed no evidence of systematic disagreement between the two specimen types (*p* = 1.000). These findings indicate complete agreement between matched plasma and urine samples within this cohort (Figure 4B).

To further assess the clinical utility of the double drop-off ddPCR assay, its performance was evaluated across matched tissue DNA, plasma cfDNA, and urine cfDNA samples. In one patient with an *FGFR3* mutation-positive primary tumor previously confirmed by NGS, the ddPCR assay successfully detected the mutation across all tested sample types. In addition, in three patients whose primary tumors were classified as *FGFR3* mutation-negative by NGS, the ddPCR assay consistently yielded negative results across the corresponding tissue, plasma, and urinary samples (Figure 4C,D).

## 4. Discussion

In BC, *FGFR3* mutations represent established molecular alterations with diagnostic and predictive relevance, particularly because they may guide the selection of patients eligible for FGFR-targeted therapies [28,29,30]. Erdafitinib has been FDA-approved as a standard-of-care treatment for patients with metastatic urothelial carcinoma harboring susceptible *FGFR2/3* genomic alterations who have progressed during or following platinum-based chemotherapy, including within 12 months of neoadjuvant or adjuvant platinum-containing chemotherapy [31]. This has created a clinical need for companion diagnostic assays that are robust, sensitive, rapid, and cost-effective. In this report, we describe a novel multiplex double drop-off ddPCR assay for the simultaneous detection of four clinically relevant *FGFR3* hotspot mutations. Beyond bladder cancer, ddPCR has been applied in several malignancies for molecular diagnosis and disease monitoring. In lung cancer, serial ctDNA analysis by ddPCR has been used to monitor treatment response and emerging resistance-associated mutations [32]. It has also enabled highly sensitive detection of minimal residual disease in leukemias and has shown potential for monitoring recurrence or treatment response in melanoma and neuroblastoma [33]. These studies support the broader clinical value of ddPCR as a complementary tool for precision oncology, although it is not itself a therapeutic approach.

The novelty of this assay, compared with previously described approaches [18,31,34,35,36], lies in its dual drop-off design, which allows the detection of four *FGFR3* hotspot mutations using only two primer pairs for the amplification of specific regions of exons 7 and 10. In the same assay, only two probes per exon are used, acting either as drop-off or reference probes depending on the mutation detected. This design enables the rapid and inexpensive detection of four *FGFR3* mutations, achieving a sensitivity of 0.2% for both target regions. Importantly, this approach preserves valuable clinical samples and minimizes the risk of false-negative results caused by low mutant DNA content, which represents one of the main limitations of sequencing-based assays. This level of sensitivity further supports the potential use of the assay for the evaluation of *FGFR3* mutations in plasma and urine samples, where the amount of available DNA is often limited.

We applied the developed double drop-off ddPCR assay to 49 liquid biopsy samples from patients with bladder cancer, including 25 plasma cfDNA samples, 24 matched urinary cfDNA samples, and four corresponding tissue samples. *FGFR3* exon 7 mutations were detected in 8.0% of plasma cfDNA samples (2/25) and 4.2% of urinary cfDNA samples (1/24), while an *FGFR3* exon 10 mutation was detected in 4.0% of plasma cfDNA samples (1/25). The mutation detection rate observed in our cohort was lower than that reported in broader urine-based screening studies. For example, Zuiverloon et al. reported *FGFR3* mutation positivity in 22.7% of 463 urine samples from patients with low-grade NMIBC, increasing to 85.7% (257/300) when urine samples from patients with known *FGFR3*-mutant tumors were analyzed [37]. This difference is likely explained by cohort selection, as *FGFR3* mutations are more frequent in low-grade NMIBC and detection rates are expected to increase when patients are preselected based on *FGFR3*-positive tumor status [38]. In contrast, our study was performed mainly in patients with locally advanced and metastatic bladder cancer. In a study including patients of similar stages to ours, albeit in a much smaller sample size (6 patients), Carrasco et al. detected *FGFR3* mutations only in 1 of 6 plasma cfDNA samples (1/6) using ddPCR [39].

Interestingly, one plasma cfDNA sample was found positive for the presence of *FGFR3* mutations in both exons 7 and 10, suggesting the coexistence of alterations within different hotspot regions of the same gene. This phenomenon of concurrent alterations is consistent with previous literature; notably, Critelli et al. [40] analyzed DNA from urinary exfoliated cells using PCR and demonstrated that 12.6% (12 out of 95) of the *FGFR3* mutation-positive samples harbored two distinct mutations.

In addition, direct comparison of the developed double drop-off ddPCR assay with NGS profiling in FFPE tissue DNA revealed an overall concordance of 74.2% between the two methodologies. FGFR3 mutations were detected in 35.5% (11/31) of FFPE tissue samples by ddPCR, whereas NGS detected *FGFR3* mutations in 9.7% (3/31) of samples. The discordant cases were all positive by ddPCR but classified as negative by NGS. This observation was further supported by McNemar’s exact test, which demonstrated a significant asymmetry in discordant classifications. These discrepancies may therefore be explained by the higher analytical sensitivity of ddPCR, which enables the detection of low-abundance mutant alleles that may not be reliably reported by sequencing-based assays. This is particularly important for liquid biopsy analysis, where the amount of available DNA is often limited and the fraction of tumor-derived mutant DNA may be very low. Although the ddPCR-positive/NGS-negative findings may reflect the lower analytical detection limit of ddPCR, the absence of independent orthogonal confirmation prevents definitive classification of these cases as true-positive variants. Therefore, the possibility of false-positive ddPCR detection cannot be excluded. Future studies should validate discordant samples using an independent method and larger, prospectively collected cohorts.

In the subset of patients (n = 4) with matched tissue DNA, plasma cfDNA, and urinary cfDNA, complete concordance was observed across all available sample types. In one patient with an *FGFR3* mutation-positive primary tumor previously confirmed by NGS, the ddPCR assay detected the mutation in tissue, plasma, and urine. Conversely, in three patients with *FGFR3* mutation-negative tumors by NGS, ddPCR results were consistently negative across matched tissue and liquid biopsy samples. Because matched tissue, plasma, and urine samples were available for only four patients, this analysis should be considered exploratory and not a clinical validation of concordance across biological compartments. These preliminary findings suggest that the assay may reliably reflect tumor *FGFR3* status across different biological materials. In agreement with these observations, Christensen et al. [41] analyzed paired plasma and urine samples (n = 23) from patients with previously confirmed tissue-positive tumors, reporting a positive correlation between tumor DNA levels and higher overall concentrations in urine. Notably, tumor DNA was invariably detected in urine whenever present in plasma, confirming the strong diagnostic interrelation between these biofluids. Furthermore, highlighting the high diagnostic sensitivity of urinary screening, Millholl et al. [42] demonstrated that a single-molecule assay identified *FGFR3* mutations in five additional urine DNA samples that had tested negative by quantitative PCR in matched tissue.

The double drop-off ddPCR assay offers several advantages, including high analytical sensitivity, rapid processing, low DNA input requirements, and simultaneous screening of four recurrent *FGFR3* mutations in tissue and liquid biopsy specimens. Its targeted design may also provide a simpler and more cost-effective alternative to broader sequencing approaches when specific hotspot mutations are of interest. Nevertheless, the assay is limited to predefined genomic regions and does not directly distinguish the specific mutation responsible for the drop-off signal. Furthermore, the relatively small number of clinical samples included in this study warrants validation in larger and more diverse cohorts.

## 5. Conclusions

We developed and analytically validated a novel multiplex double drop-off ddPCR assay for the simultaneous detection of four clinically relevant *FGFR3* hotspot mutations located in exons 7 and 10. The assay demonstrated high analytical specificity, reproducibility and sensitivity, with a limit of detection of 0.2%, supporting its suitability for the analysis of low-input clinical samples. Compared with NGS profiling, the assay identified additional low-abundance *FGFR3* mutations that were below the reporting threshold of sequencing, highlighting the increased sensitivity of ddPCR for detecting rare mutant alleles. Application of the assay to plasma and urinary cfDNA from patients with bladder cancer further demonstrated its feasibility in liquid biopsy specimens and showed concordant results between matched biofluids in the available paired samples. Future studies should validate this assay in larger and clinically well-characterized cohorts and assess its utility for longitudinal monitoring, evaluation of treatment response, and early detection of disease progression. Further standardization and prospective clinical validation will also be required before its implementation in routine molecular testing.

## Data Availability

The data presented in this study are available on request from the corresponding author.

## Supplementary Materials

The following supporting information can be downloaded at: https://www.mdpi.com/article/10.3390/cancers18162634/s1, Table S1: Characteristics of 25 patients included in the study at the time of sample collection; Table S2: Primers & Probes for the double ddPCR FGFR3 assay; Table S3: Synthetic oligonucleotides; Figure S1: (A) Intra-assay repeatability evaluated using mutant positive controls for *FGFR3* exon 7 (n = 4 replicates) and exon 10 (n = 3 replicates) within a single analytical run. (B) Inter-assay reproducibility was evaluated across independent ddPCR runs for exon 7 (n = 4 runs) and exon 10 (n = 3 runs). Individual data points represent absolute quantification values (copies/µL). Corresponding coefficients of variation (CV %) are indicated above each data series; Figure S2: Validation of double Drop-Off ddPCR Assay for the S249C mutation via linear regression against the singleplex assay (Pearson’s r = 0.991, *p* = 0.009, R^2^ = 0.98). Data points represent absolute quantification values expressed in copies/µL. Agreement between methods was further evaluated using Bland–Altman analysis. Data points represent absolute quantification values expressed in copies/µL.

## Abbreviations

The following abbreviations are used in this manuscript:

WT	Wild-type
gDNA	Genomic DNA
cfDNA	Cell-free DNA
FGFR3	Fibroblast Growth Factor Receptor-3
LOB	Limit of Blank
LOD	Limit of Detection
HDs	Healthy donors
ddPCR	Droplet Digital PCR
BC	Bladder cancer
UC	Urothelial cancer
LB	Liquid Biopsy
MIBC	Muscle-invasive bladder cancer
NMIBC	Non-muscle-invasive bladder cancer
TURB-T	Transurethral Resection of Bladder Tumors
NGS	Next-generation Sequencing
MAF	Mutant Allele Frequency
FFPE	Formalin-fixed Paraffin-embedded
