# Supplementary material for: Development and Broad Application of a Double Drop-Off ddPCR Assay for Simultaneous Detection of Four FGFR3 Mutations in Tissue and Liquid Biopsy Samples
Source: Cancers (Basel). 2026 Aug 14;18(16):2634. doi: 10.3390/cancers18162634 (PMC13510398; doi:10.3390/cancers18162634)
Supplement: Supplementary file 1 [file cancers-18-02634-s001.zip › cancers-4467682-supplementary.pdf]

Table S1: Characteristics of 25 patients included in the study at the time of sample collection

Characteristic	n
Age (median)	65
Gender<br>M<br>F	 19<br>6
Primary site<br>Bladder<br>UUTC	 23<br>2
Stage at sample collection<br>NMIBC<br>Non-metastatic MIBC<br>Metastatic	 3<br>12<br>10
Prior therapy for urothelial cancer within a month prior to sample collection<br>None<br>Enfortumab vedotin/Pembrolizumab<br>BCG intravesical/Pembrolizumab<br>Gemcitabine cisplatin	 18<br>3<br>1<br>3

Table S2: Primers& Probes for the double ddPCR FGFR3 assay

**Primers**	**Sequence**
Exon 7	Forward: 5'-GCCCCTGAGCGTCATCTG -3'
	Reverse: 5'- CGTGCTTGAGCCACTGGAT -3'
Exon 10	Forward: 5'- TCTTTGCAGCCGAGGAGGA -3'
	Reverse: 5'-AGGCGGCAGAGCGTCACA -3'

**Probes exon 7**	**Sequence**
Drop-off	5'-/56-FAM/ ACAGAGCGCTCCCCGCACC /3BHQ_1/-3'
Reference	5'-/5HEX/ TTCCACTGCAAGGTGTACAGTGACG /3BHQ1/-3'

**Probes exon 10**	**Sequence**
Drop-off	5'-/56-FAM/ AGGCGGGCAGTGTGTATGCAGG /3BHQ_1/-3'
Reference	5'-/5HEX/CTTCTTCCTGTTCATCCTGGTGGTGG /3BHQ_1/-3'

Table S3: Synthetic oligonucleotides

**FGFR3 WT(1):**<br>5’TGAGGGAGGGGGTGGCCCCTGAGCGTCATCTGCCCCCACAGAGCGCTCCCCGCACCGGCC<br>CATCCTGCAGGCGGGGCTGCCGGCCAACCAGACGGCGGTGCTGGGCAGCGACGTGGAGTTC<br>CACTGCAAGGTGTACAGTGACGCACAGCCCCACATCCAGTGGCTCAAGCACGTGGAGGTGA<br>ATGGCAGCAA 3’
**S249C:**<br>5’TGAGGGAGGGGGTGGCCCCTGAGCGTCATCTGCCCCCACAGAGCGCTGCCCGCACCGGCC<br>CATCCTGCAGGCGGGGCTGCCGGCCAACCAGACGGCGGTGCTGGGCAGCGACGTGGAGTTC<br>CACTGCAAGGTGTACAGTGACGCACAGCCCCACATCCAGTGGCTCAAGCACGTGGAGGTGA<br>ATGGCAGCAA 3’
**R248C:**<br>5’TGAGGGAGGGGGTGGCCCCTGAGCGTCATCTGCCCCCACAGAGTGCTCCCCGCACCGGCC<br>CATCCTGCAGGCGGGGCTGCCGGCCAACCAGACGGCGGTGCTGGGCAGCGACGTGGAGTTC<br>CACTGCAAGGTGTACAGTGACGCACAGCCCCACATCCAGTGGCTCAAGCACGTGGAGGTGA<br>ATGGCAGCAA 3’
**FGFR3 WT (2):**<br>5’AACGCCCATGTCTTTGCAGCCGAGGAGGAGCTGGTGGAGGCTGACGAGGCGGGCAGTGT<br>GTATGCAGGCATCCTCAGCTACGGGGTGGGCTTCTTCCTGTTCATCCTGGTGGTGGCGGCTG<br>TGACGCTCTGCCGCCTGCGCAGCCCCCCCAAGAAA3’
**Y373C:**<br>5’AACGCCCATGTCTTTGCAGCCGAGGAGGAGCTGGTGGAGGCTGACGAGGCGGGCAGTGT<br>GTGTGCAGGCATCCTCAGCTACGGGGTGGGCTTCTTCCTGTTCATCCTGGTGGTGGCGGCTG<br>TGACGCTCTGCCGCCTGCGCAGCCCCCCCAAGAAA3’
**G370C:**<br>5’AACGCCCATGTCTTTGCAGCCGAGGAGGAGCTGGTGGAGGCTGACGAGGCGTGCAGTGTG<br>TGTGCAGGCATCCTCAGCTACGGGGTGGGCTTCTTCCTGTTCATCCTGGTGGTGGCGGCTGT<br>GACGCTCTGCCGCCTGCGCAGCCCCCCCAAGAAA3’

Supplementary Figure S1

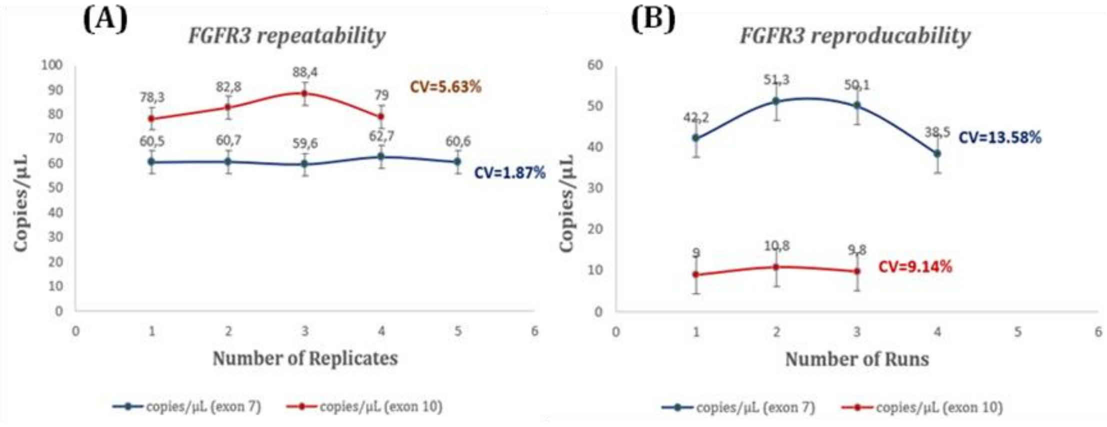

Supplementary Figure S2

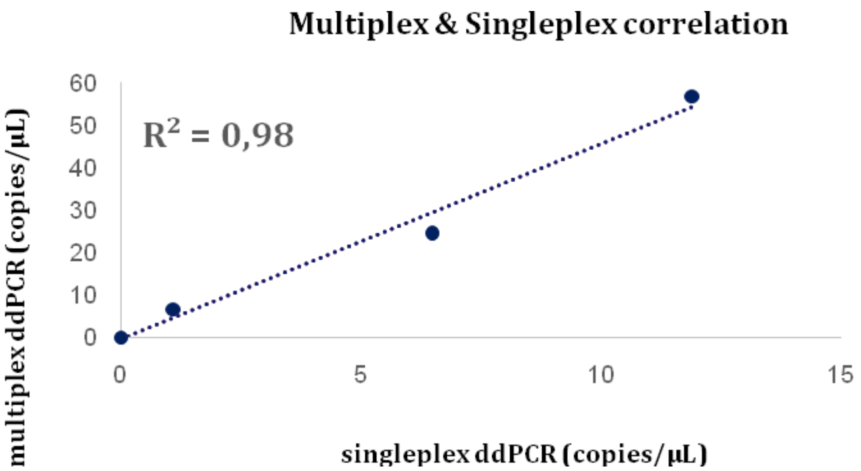

Multiplex & Singleplex correlation
R^2 = 0,98
60
50
40
30
20
10
0
0
5
10
15
multiplex ddPCR (copies/µL)
singleplex ddPCR (copies/µL)
